# Bioinformatics of Cancer ncRNA in High Throughput Sequencing: Present State and Challenges

**DOI:** 10.3389/fgene.2012.00287

**Published:** 2012-12-17

**Authors:** Natasha Andressa Nogueira Jorge, Carlos Gil Ferreira, Fabio Passetti

**Affiliations:** ^1^Bioinformatics Unit, Clinical Research Coordination, Instituto Nacional de CâncerRio de Janeiro, Brazil; ^2^Clinical Research Coordination, Instituto Nacional de CâncerRio de Janeiro, Brazil

**Keywords:** bioinformatics, high throughput sequencing, cancer, non-coding RNA, gene expression

## Abstract

The numerous genome sequencing projects produced unprecedented amount of data providing significant information to the discovery of novel non-coding RNA (ncRNA). Several ncRNAs have been described to control gene expression and display important role during cell differentiation and homeostasis. In the last decade, high throughput methods in conjunction with approaches in bioinformatics have been used to identify, classify, and evaluate the expression of hundreds of ncRNA in normal and pathological states, such as cancer. Patient outcomes have been already associated with differential expression of ncRNAs in normal and tumoral tissues, providing new insights in the development of innovative therapeutic strategies in oncology. In this review, we present and discuss bioinformatics advances in the development of computational approaches to analyze and discover ncRNA data in oncology using high throughput sequencing technologies.

## Introduction

The ENCODE project discovered that most of the human genome is transcribed, but only a tiny fraction of human DNA encode for proteins (ENCODE Project Consortium et al., 2007; Elgar and Vavouri, 2008). The remaining transcriptome is defined as non-coding RNA (ncRNA) and is divided into distinct classes, each of them with its own three-dimensional folding and presenting a specific function. Some ncRNA classes are known for years, such as ribosomal and transport RNAs (essential to translation); small nucleolar RNAs (snoRNAs; biogenesis and control of ribosome activity); and small nuclear RNAs (to promote splicing of pre-mRNAs). Recently, additional ncRNA classes have been described and shown to be able to repress gene expression (microRNAs, miRNA); to regulate cellular proliferation, apoptosis (small interfering RNAs, siRNAs), and imprinting (long non-coding RNAs, lncRNA); and also to inhibit transposon and DNA methylation (PIWI-interacting RNAs, piRNA; for a detailed description of the known ncRNAs, see Eddy, 2001; Mitra et al., 2012).

The most studied ncRNA class in oncology is miRNA. These small RNAs have on average 22 nucleotides in length and mediate gene silencing by partially paring with specific regions of messenger RNAs (mRNA) to prevent its translation (Wu et al., 2012). The miRNA target genes are usually related to fundamental cellular processes like proliferation, differentiation, apoptosis, and development (Schulte et al., 2010). Aberrations in miRNAs expression levels have been extensively studied in several types of cancer as they may act as tumor suppressor genes or oncogenes (Meiri et al., 2010).

Additionally, two ncRNA classes with special attention in studies in oncology are lncRNA and piRNA. The lncRNAs are more than 200 nucleotides long and although most of them have not been fully characterized, they have been related to the regulation of several cellular processes such as epigenetics, differentiation, proliferation, and nuclear import (Tahira et al., 2011). Recent studies reported alterations in different lncRNAs in several types of cancer (Reis et al., 2004; Guffanti et al., 2009; Cheng et al., 2011; Cui et al., 2011; Esposito et al., 2011; Prensner et al., 2011; Tahira et al., 2011; Yang et al., 2012a,b). The piRNA class has also been related to have a possible involvement in the biogenesis of cancer. The piRNAs interact with PIWI proteins in order to promote silencing of transposable elements and maintain DNA integrity (Cheng et al., 2011).

Since 1977, when the first genome was sequenced, the DNA sequencing technology has been evolving to higher throughput and lower cost (Kircher and Kelso, 2010). Current high throughput sequencing (HTS), also known as next-generation sequencing, provides the opportunity to obtain a more accurate profiling with higher resolution, increased throughput, sequencing depth, and low experimental complexity (Prensner et al., 2011; Zhou et al., 2011). One characteristic of this technology is the amount of data produced, making methods in bioinformatics essential for its analysis.

Bioinformatics emerged as a multidisciplinary discipline which aimed to analyze biological data using programming techniques and the computational processing power. The first studies in Bioinformatics were performed in the early 1960s, when the first computational approaches were used to address gene and protein sequences (for a time line review, see Hagen, 2000). The term bioinformatics was coined by Hesper and Hogeweg (1970) as “the study of informatics processes in biotic systems” (Hogeweg, 2011). However, after the emergence of high throughput methods in molecular biology and the establishment of the Human Genome Program in 1990, the definition of bioinformatics has shifted to assist in the management, storage, visualization, and analysis of large amounts of data. In conjunction to the development of bioinformatics tools, many molecular biology techniques were created in the last two decades such as qPCR, microarray, tilling array and SAGE, which permitted to quantify gene expression. A large number of studies have been taken using molecular biology techniques to produce large amounts of raw data and bioinformatics tools to assist the biological interpretation of the findings. An example of the importance of bioinformatics to the science was the announcement of the draft of the human genome in 2001, which was presented after the development of a computational tool to assemble the unsorted fragments of the human genome (Kent and Haussler, 2001; Lander et al., 2001).

**Figure 1:**
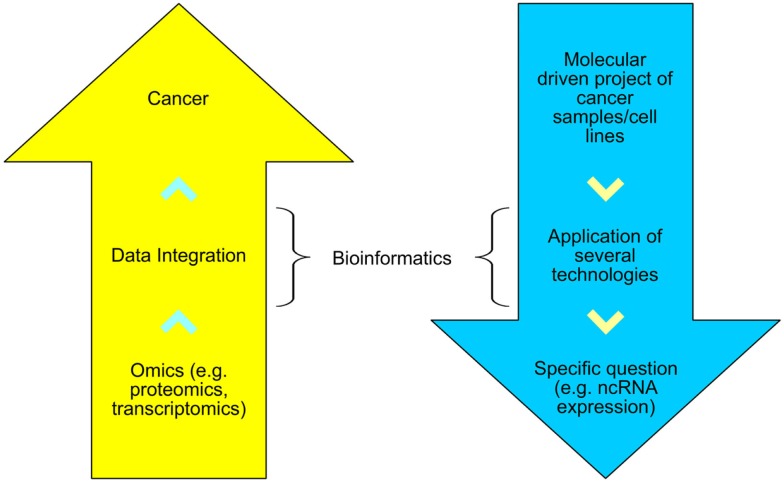
**The disease-oriented and methodological-driven types of research assisted by bioinformatics**.

As depicted in Figure 1, bioinformatics can assist two types of research: disease-oriented (e.g., cancer) and methodologically driven (e.g., HTS). In the former, several technologies can be used to study distinct biological patterns and then a systems biology approach is taken to assist in the comprehension of cancer. In the latter, an unique molecular biology technique is used to answer a specific interrogation, for example, the expression pattern of human genes after a group of patients received a standard treatment against a specific cancer type.

In this review, we present some examples of ncRNA discovered, its potential to be used as cancer biomarkers and the role and challenges in bioinformatics to analyze HTS data.

## Why Studying Non-Coding RNAs in Cancer?

Calin et al. (2002) documented the first differentially expressed ncRNA in cancer samples. The small RNAs *miR-15* and *miR-16* were described to be deleted or down regulated in more than half of the patients with Chronic Lymphocytic Leukemia (CLL) and B-cell CLL. The absence of those genes led to an over expression of the *Bcl-2* gene, preventing apoptosis. Two years later, additional data revealed that some miRNAs genes are located at fragile and frequently altered sites in cancer, including regions with amplifications, loss of heterozygosity, or breakpoints (Calin et al., 2004). Since then, several other reports have presented alterations related to ncRNAs in different cancer samples.

One of the first approaches to associate ncRNA and oncology was performed by Mishra et al. (2007). The authors evaluated polymorphisms in the human dihydrofolate reductase (DHFR) mRNA binding site for *miR-24*. As result, the polymorphism led to the loss of *miR-24* function and resulted in *DHFR* overexpression, increasing resistance to chemotherapy. Among miRNAs, the oncogene *miR-21* has been extensively studied (Dillhoff et al., 2008; Frankel et al., 2008; Krichevsky and Gabriely, 2009; Li et al., 2009a,b; Rabinowits et al., 2009; Ribas et al., 2009; Seike et al., 2009; Wickramasinghe et al., 2009; Iliopoulos et al., 2010). This miRNA appears over expressed in different tumor samples and targets *PTEN*, *PDCD4*, *TPM1*, and *Maspin* human genes, promoting growth, migration, and invasion in different tumor types (Zhu et al., 2008).

Regarding lncRNAs, recently, a single nucleotide polymorphism located in the *ANRIL* gene was associated with the number of plexiform neurofibromas in neurofibromatosis type 1 patients. Moreover, one of its allele was associated with low levels of *ANRIL*, suggesting a relation between the *ANRIL* and the susceptibility to plexiform neurofibromas (Pasmant et al., 2011). In addition, in a recent review, Gustschner and Diederichs (2012) were able to link cellular processes influenced by lncRNAs to the hallmarks of cancer.

Several studies associating cancer and ncRNA aim to discover molecular signatures for diagnosis and prognosis. In this direction, cancer biomarkers are molecular features that are produced either by the tumor or by the host as a response due to the change of the default cell metabolism. Examples of possible biomarkers are mutations and alterations in gene expression and epigenetics (for a deep view of cancer epigenetics, see Brait and Sidransky, 2011). The identification of specific cancer biomarkers may provide parameters for cancer early detection, diagnosis, prognosis, prediction of response to anticancer treatments, prediction of recurrence, and identification of putative drug targets. However, due to cancer complexity, it has been recently suggested that single biomarker may not be adequate for clinical practice and it is suggested to use a set of biomarkers in a panel (Tainsky, 2009). The study of Hennessey et al. (2012) compared the miRNA expression profile in the serum of non-small cell lung cancer (NSLC) patients and healthy individuals. The authors proposed the combination of the expression levels of *miR-15b* and *miR-27b* would be able to discriminate the healthy and the sick individuals. Another study in NSLC was performed by Chen et al. (2012) in which it is suggested a 10 miRNA panel to differentiate tumor types. Wu et al. (2012) analyzed the serum of 42 breast cancer patients and were able to detect more than 800 circulating miRNAs and associate them with tumor status. The low levels of miRNA *miR-375* and high levels of miRNA *miR-122* have been suggested as biomarkers for predicting metastasis in early patients. In this direction, Liu et al. (2011) compared the expression of miRNAs in the serum of 20 patients with gastric cancer against 20 normal samples. Among the 19 over expressed miRNA identified, the *miR-1*, *miR-20a*, *miR-27a*, *miR-34*, and *miR-423-5p* have been identified as potential biomarkers for gastric cancer diagnostics and tumor profiling.

Another aspect of ncRNA and cancer is the possibility to associate them with drug resistance. A very large effort to comprehend the role of drug activity and resistance in cancer cell lines was performed by Liu et al. (2010). The microarray technology has been used to evaluate the mRNA and miRNA expression profiling of the 60 cancer cell lines of the National Cancer Institute Developmental Therapeutics Program, also known as the NCI-60 panel. The authors used bioinformatics approaches to analyze and cluster some cell groups according to their tissue of origin and to associate the levels of mRNAs and miRNAs with sensitivity or resistance to many drugs routinely used in the clinic. To facilitate the visualization of the data produced, the authors developed the CellMiner, a web based tool very useful to clinicians and researchers from basic to applied research (Reinhold et al., 2012).

The aforementioned studies exemplify how miRNA are involved in cancer development and progression. Another advantage of analyzing small ncRNA profile in cancer regards the distinct types of samples may be use to study it, from fresh tissues, body fluids (including blood, urine, and saliva), and formalin-fixed, paraffin-embedded (FFPE) tissues (Lussier et al., 2012). Therefore, the study of ncRNAs and its expression profiling in cancer cells may help understand the mechanisms of the disease and improve diagnostics and prognostics by personalizing cancer treatment (Hu et al., 2010).

## Why Using HTS for ncRNA Profiling in Cancer?

**Figure 2:**
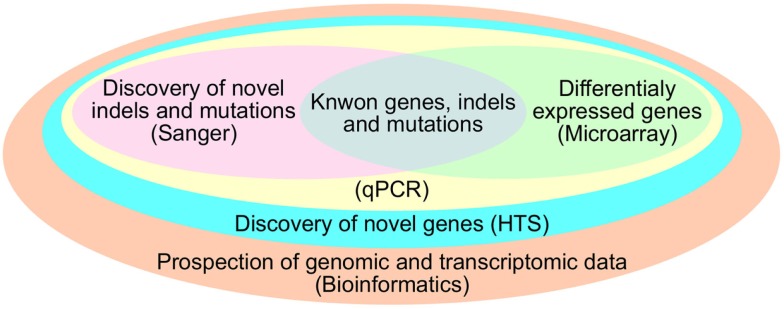
**Advantages of Bioinformatics and HTS over other techniques**.

The most common approach used to study ncRNA is to first produce large-scale profiling on microarray followed by validation by more specific techniques such as microarray with fewer probes or multiplexed RT-PCR. Regarding ncRNAs, miRNA microarrays provide an overview of the set of miRNAs in a sample and can be further validated by northen blot, Rnase protection assay, primer extension assay, quantitative RT-PCR, and *in situ* hybridization (Tainsky, 2009). However, with the advent of HTS technology, it is possible not only to infer the expression level of ncRNA, but also to detect uncharacterized ones. Another advantage of HTS over other existing expression profiling technologies is the fact that the process requires no previous information about the transcripts that will have its expression quantified (Isakov et al., 2012). This characteristic of HTS is suggestive for its use in the quantification of the heterogeneous transcriptome of cancer (Meyerson et al., 2010). Distinct from other techniques, HTS does not use specific or random probes, instead, the RNA molecules from the sample are linked to adaptors and amplified by PCR (McCormick et al., 2011), permitting the sequencing of the exact transcript on a single nucleotide resolution (Zhou et al., 2011). This step allows the identification of variations in length or composition, deletions, duplications, low abundant, and novel transcripts present in cancer samples (Meyerson et al., 2010). Figure 2 depicts some advantages of HTS over other techniques and how bioinformatics is essential to analyze them.

A comparison between the expression profile using HTS and microarray was performed by Weng et al. (2010). The authors used HTS technology to evaluate the profile of small RNAs in three paired clear cell renal cell carcinoma (ccRCC) FFPE samples and performed miRNA microarray and RT-PCR to validate the results from the former. Besides the known miRNA genes, the HTS experiments were able to reveal million of short sequences that included sequences from snoRNAs, sRNA, snRNA, tRNAs, rRNAs, introns, exons, and several others, including unknown nucleotide sequences. Bioinformatics techniques were used to cluster the miRNA detected and to distinguish between tumor and normal samples. The miRNA microarray were able to detect up to 453 miRNAs, while the HTS could identify up to 598 miRNAs and both platforms showed correlated expression levels that were validated by RT-PCR in seven randomly chosen altered miRNAs. As can be observed, HTS let to the quantification of 145 additional ncRNAs not present in the microarray experiment.

Several ncRNA HTS studies revealed putative novel ncRNAs (Jima et al., 2010; Keller et al., 2011; Prensner et al., 2011). Deep sequencing of the enriched Poly(A) transcriptome was used to evaluate the expression of both protein coding and lncRNAs in cancer samples by Prensner et al. (2011) in 102 prostate tissues and cell lines, including normal samples and benign, localized, and metastatic samples. The authors were able to describe the novel lncRNA *PCAT-1*, over expressed in metastatic samples. Further experiments pointed it as a prostate specific regulator of cell proliferation that targets the Policomb Repressive Complex 2 (*PRC2*). Jima et al. (2010) evaluated small ncRNAs in normal and malignant B cells. The authors proposed a panel of known and novel miRNAs to distinguish between the subgroups of lymphoma and found that one previously annotated miRNA cluster has its expression levels inversely correlated with its putative targets *SMAD2* and *SMAD3*, known mediators of the transforming growth factor-β (TGF-β) signaling pathway. Keller et al. (2011) evaluated the miRNAs differentially expressed in the blood of NSLC patients and found some unknown miRNAs, including novel mature forms from known precursors.

Another example of HTS as tool to the identification of novel small ncRNA class is found in the study of Meiri et al. (2010). The authors used HTS to evaluate the miRNA transcriptome of 23 solid tumor samples, including breast, bladder, colon, and lung. They discovered 49 novel miRNA and sequence variants with different expression patterns among the samples and identified a novel class of small ncRNAs derived from Y-RNAs and endogenous siRNAs.

Most of the HTS studies published so far have tried to identify miRNA to use as diagnostic or prognostic biomarkers in solid tumors or in circulation. The two studies by Wu et al. (2012) and Liu et al. (2011) referred to in the previous section used HTS to infer their candidate biomarkers. Martens-Uzunova et al. (2012) and Ryu et al. (2011) went further. Martens-Uzunova et al. (2012) used the miRNA expression found in one organ-confined and one metastatic lymph node tumor samples of prostate cancer to create a miR-classifier that was able to correctly distinguish 89% of the prostate cancer cell samples. Besides miRNA, the experiment was able to find snoRNAs and tRNAs with altered expression levels and novel miRNA with very low counts. Ryu et al. (2011) applied a bioinformatics approach to validate the novel miRNAs in breast cancer cell lines. The authors obtained 189 putative novel miRNAs, considering thermodynamics stability, presence of complementary sequences, and phylogenetic conservation.

There are several HTS platforms commercially available, each with its own characteristics such as data throughput, read length, error rate, and price (Zhou et al., 2011). Therefore, the choice of the platform to be used must be according to its characteristics and the needs of the experiment. Kircher and Kelso (2010) reviewed the sequencing technologies of some HTS platforms and Toedling et al. (2012) present the comparison of different sequencing protocols and the results obtained. The authors recommend comparing data generated only by the same protocol.

## How Computational Procedures can Aid ncRNA HTS Profiling?

**Figure 3:**
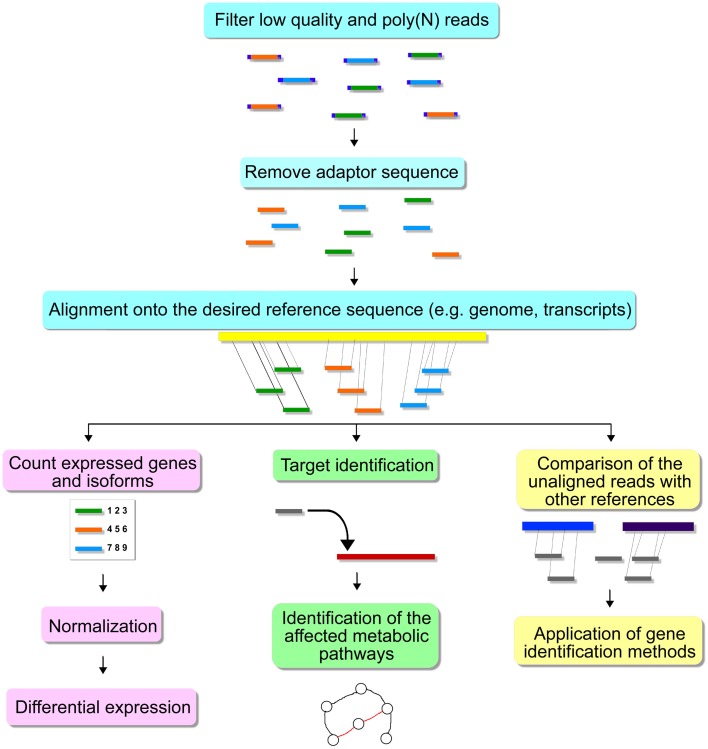
**Steps for HTS analysis**.

High throughput sequencing experiments generate a large amount of data, hence bioinformatics methods are necessary for the proper storage, visualization, and analysis. After sequencing, one or more text files are produced in the fasta, fastq or csfasta, and qual formats, depending on the equipment settings and platform used. These files contain the nucleotides sequenced for each read and a quality score for each base/color call (Isakov and Shomron, 2011). Usually, the sequencer manufacturer provides software able to process this data in the very beginning steps toward publication. In this section, we will discuss available independent tools for each step of the downstream analysis. Figure 3 shows some of the steps for HTS analysis.

Among the sequenced data, it is common to find reads with miscalled bases, unidentified bases, poor quality, and adaptor contamination. Those artifacts must be removed before alignment to avoid wrong mapping and also to save computational time (Patel and Jain, 2012).

For the removal of low quality reads and unidentified bases, some authors use their own script as described, for example, by Meiri et al. (2010). However, other studies use public available toolkits, like Fastx-toolkit (Gordon and Hannon, unpublished) and QC Toolkit (Patel and Jain, 2012). The aforementioned tools are a collection of programs for processing short reads fastq and fasta files and reporting the quality of sequencing run, filtering reads for their quality, and removing unknown nucleotides.

If the aim is to sequence short RNAs (sRNAs), most probably the size of the desired sRNA is smaller than the read’s length (Martin, 2011). In this case, a subsequence of the adaptor used in the sequencing process will be present in the final result and, because it does not belong to the sequenced genome, it must be removed (McCormick et al., 2011). Both of the toolkits mentioned above can remove those sequences. Other tools include the Cutadapt program (Martin, 2011), the Bioconductor’s package for short read processing called Biostrings (Pàges et al., 2012) and the aligners Novoalign (Hercus, 2008) and SOAP (version 1; Li et al., 2008). Table 1 presents some preprocessing alignment tools. The Bioconductor’s packages Biostrings (Pàges et al., 2012) and ShortRead (Morgan et al., 2009) together can assess the quality and remove adaptor sequence from fasta and fastq files, but they require user knowledge of programming language R and Bioconductor. The Cutadapt algorithm can remove the adaptor sequence from the reads obtained by the major sequencing platforms, but, differently from the aforementioned algorithms, it cannot access or filter low quality reads (Martin, 2011). Regarding the mentioned aligners, its adaptor removal propriety is linked to the alignment algorithm; therefore they cannot be applied if the user wishes to use another alignment tool.

**Table 1:** **Preprocessing alignment tools**.

Name	Site	Description	Authors
Fastx-toolkit	http://hannonlab.cshl.edu/fastx_toolkit/	FASTA/FASTQ file processing	Gordon and Hannon (unpublished)
QC tools	http://www.nipgr.res.in/ngsqctoolkit.html	Ilumina and Roche 454 FASTQ file processing	Patel and Jain (2012)
Cutadapt	http://code.google.com/p/cutadapt/	Removes adapter sequence	Martin (2011)
ShortRead	http://bioconductor.org/packages/2.10/bioc/html/ShortRead.html	FASTA/FASTQ file processing	Morgan et al. (2009)
Biostrings	http://bioconductor.org/packages/2.10/bioc/html/Biostrings.html	String objects representing biological sequences, and matching algorithms	Pàges et al. (2012); R package version 2.24.1

The next step of the analysis is aligning sequence reads onto the genome of the reference organism. This can be a computationally demanding task due to the great volume of short sequences produced and also nucleotide and structural variance, sequencing errors, RNA editing, and epigenetic modifications (Isakov and Shomron, 2011), especially for the traditional alignment tools (Lee et al., 2011). Hence, a new generation of short read aligners has been developed, saving computational time by indexing the read sequences, or the genome prior alignment (Lee et al., 2011). Several aspects of the aligner must be considered: memory and time requirements and limitations, and how the tool is adequate to the task (Isakov and Shomron, 2011). For instance, many short read aligners can be programmed to return the results of the reads whose first part perfectly matches the reference genome, which allows to search for potential isoforms of miRNA (Motameny et al., 2010). In this direction, the Novoalign software has a special option to align miRNA in which it searches for regions complementary to the reads near the mapped loci (Hercus, 2008). Most sequence aligners generate results in the sam file format which can be processed by the SAMtools kit (Li et al., 2009c; Isakov and Shomron, 2011). One thing worth noticing is that when a short sequence is aligned to a large and complex genome with repetitive regions, such as the human genome, is expected to find reads mapped in multiple locations in the genome (McCormick et al., 2011). Most software does not report such results as default, resulting in the loss of some sequences (Motameny et al., 2010). Other strategies to manipulate such reads are to divide their count by all putative loci and their estimate a proportion according to the levels of uniquely mapped reads in neighbor loci (McCormick et al., 2011). Some alignment tools for HTS data are shown in Table 2 and were evaluated by Ruffalo et al. (2011).

**Table 2:** **Alignment tools**.

Name	Site	Authors
Soap	http://soap.genomics.org.cn/soapaligner.html	Li et al. (2008)
Bwa	http://bio-bwa.sourceforge.net/	Li and Durbin (2009)
Bowtie	http://bowtie-bio.sourceforge.net/index.shtml	Langmead et al. (2009)
Novoalign	http://www.novocraft.com/main/index.php	Hercus (2008)

As important as the aligner, is the database to map the processed reads. There are several genome and ncRNA databases available, but the most commonly used sequence databases for studying cancer are the following: the human genome hg18 assembly provided by the UCSC Genome Bioinformatics group (Dreszer et al., 2012), miRBase (Kozomara and Griffiths-Jones, 2011) and Rfam (Gardner et al., 2011). It is important to notice that the human genome sequence in the hg18 version provided through the UCSC Genome Browser website is identical to the NCBI36 version. Table 3 exemplifies some of these databases.

**Table 3:** **Sequence databases**.

Name	Site	Description	Authors
UCSC hg18/NCBI36	http://genome.ucsc.edu/	Human genome sequence	International Human Genome Sequencing Consortium
ncRNA.org	http://www.ncrna.org/	ncRNA database sequence	Mituyama et al. (2009)
miRBase	http://www.mirbase.org/	miRNA database sequence	Kozomara and Griffiths-Jones (2011)
Rfam	http://rfam.sanger.ac.uk/	ncRNA database sequence	Gardner et al. (2011)

Regarding ncRNA analysis, it is important to use annotation databases having information regarding the annotation of prediction and experimentally defined ncRNAs. The UCSC Table Browser provides open accesses to high quality human genome annotation including alignment of RefSeq genes, mRNAs and EST from GenBank and also other gene and gene prediction tracks such as Ensembl Genes (Karolchik et al., 2004). Currently, this tool is under migration to the latest version of the human genome sequence (hg19/NCBI37; Dreszer et al., 2012). One another important source of annotation files for studying ncRNA is ncRNA.org, which is part of the Functional RNA database and is an extended mirror of the UCSC Genome Browser. NcRNA.org displays information about functional ncRNAs and associated elements in the hg17 and hg18 versions of the human genome (Mituyama et al., 2009). Another frequently database used in studies in oncology and HTS is the miRBase (Kozomara and Griffiths-Jones, 2011). This database is the primary source for miRNA sequence and annotation. The miRBase effort has the objective to provide curated nomenclature scheme for known and novel miRNAs, to act as central repository for mature and precursor miRNA sequence and also to provide access to the primary evidence that supports miRNA annotations. Another database used in researches that go beyond the miRNA family is named Rfam. This database maintains automated and curated sequences, alignments, secondary structure, and annotations of several ncRNAs families. Each family represents a set of RNA sequences that share a common ancestral (Gardner et al., 2011).

All the aforementioned tools require Linux and programming knowledge from the end user. Aiming to assist small to medium bioinformatics research groups to analyze miRNA HTS, several pipelines have been developed for processing raw files, identify novel transcripts, calculate differential expression, and provide fast annotation of genomic coordinates and single nucleotide variations (revised by Li et al., 2012; Table 4). One exception is the RandA pipeline (Isakov et al., 2012), that uses the whole Rfam database, and can be applied to different ncRNAs. Segtor (Renaud et al., 2011) is another tool that works to assist in one important step in the biological interpretation effort of every HTS experiment. Segtor allows the fast annotation of sequences from a given HTS experiment and provide a list of ncRNA genes affected by multiple types of nucleotide polymorphisms.

**Table 4:** **Pipelines for HTS analysis**.

Name	Site	Description	Authors
miRExpress	http://mirexpress.mbc.nctu.edu.tw/	miRNA profiling	Wang et al. (2009)
RandA	http://ibis.tau.ac.il/RandA/	ncRNA profiling and differential expression	Isakov et al. (2012)
mirAnalyzer	http://bioinfo2.ugr.es/miRanalyzer/miRanalyzer.php	miRNA profiling and gene discovery	Hackenberg et al. (2009)
miRNAkey	http://ibis.tau.ac.il/miRNAkey/	miRNA profiling and differential expression	Ronen et al. (2010)

One of the advantages of HTS over other profile techniques resides in the fact that its quantification is based on how many reads were mapped in the same region/transcript. However, the read count is subject to sample and experimental variation, therefore, they must be normalized to be compared to other samples (Datta et al., 2010). There are several normalization methods, like linear total count scaling, quantile-based, trimmed mean of *M* value, two-step non-linear regression and others, each with its own advantages and disadvantages (McCormick et al., 2011). One of the most common normalization methods is to compute the RPKM (reads per kilobase per million) of each unique reads (Motameny et al., 2010). Some of the mentioned methods can be applied using the Bioconductor’s package easyRNASeq (Delhomme et al., 2012). This process must not include the sequencing errors that passed the initial filters and it is also recommended to remove reads with low counts (Motameny et al., 2010).

After normalization, the appropriate statistical method can be applied to find differentially expressed ncRNAs. Microarray is a method widely used for large-scale quantification of gene expression. However, raw data from microarray and HTS differ because the former provides continuous values and the latter discrete values for measuring gene expression. Hence, well-established statistical methods used for the detection of differentially expressed genes in microarray data cannot be applied for HTS studies. Some examples of packages and softwares for HTS analysis are the Bioconductor’s packages DESeq (Anders and Huber, 2010), EdgeR (Robinson et al., 2010), based on the negative binomial distribution, and baySeq (Hardcastle and Kelly, 2010), which uses a statistical Bayesian approach. Some authors also prefer to use variations of the Poison’s distribution like the Two-Stage Poison Model (Auer and Doerge, 2011). Recently, some articles were published comparing the performance of some of the aforementioned differential expression Bioconductor packages and other softwares based on simulated and real data (Kvam et al., 2012; Robles et al., 2012; Vijay et al., 2012). Table 5 presents some Bioconductor’s packages for normalization or differential expression analysis of HTS data.

**Table 5:** **Bioconductor’s packages for normalization and differential expression of HTS data**.

Name	Site	Description	Authors
easyRNASeq	http://bioconductor.org/packages/2.10/bioc/html/easyRNASeq.html	Count summarization and normalization for RNA-seq data	Delhomme et al. (2012)
DESeq	http://bioconductor.org/packages/2.10/bioc/html/DESeq.html	Differential gene expression analysis based on the negative binomial distribution	Anders and Huber (2010)
edgeR	http://bioconductor.org/packages/2.10/bioc/html/edgeR.html	Empirical analysis of digital gene expression data in R	Robinson et al. (2010)
baySeq	http://www.bioconductor.org/packages/release/bioc/html/baySeq.html	Normalization and differential gene expression by Bayesian methods	Hardcastle and Kelly (2010)

It is interesting to further validate any novel transcripts discovered. Computational and experimental techniques for gene finding are difficult to be applied to ncRNAs, due to their specific function and the fact that they do not have the same characteristics as the well known protein coding genes (Mendes et al., 2009). Concerning ncRNAs, most of the gene finding tools is directed to miRNA genes (revised by Oulas et al., 2011). A tool constructed specially to validate novel miRNAs found by HTS experiments is mirDeep (Friedländer et al., 2008; Table 6). This tool searches for reads that form the precursor miRNA and uses the folding algorithm of the Vienna package to evaluate the possibility of a hairpin structure (Friedländer et al., 2008). As mentioned, the structure of ncRNA families is well conserved and is usually used to assist as an additional step toward confirming a new or a known ncRNA.

**Table 6:** **miRNA gene discovery for HTS**.

Name	Site	Authors
miRDeep	http://www.mdc-berlin.de/en/research/research_teams/systems_biology_of_gene_regulatory_elements/projects/miRDeep/index.html	Friedländer et al. (2008)

There are several folding algorithms to predict RNA secondary structure (Table 7). Among the most well known are the ViennaRNA package (Lorenz et al., 2011), Mfold (Zuker, 2003) and Rfold (Kiryu et al., 2008). The ViennaRNA package uses thermodynamic parameters and dynamic programming to predict the secondary structure. It also provides information about centroid and maximum expected accuracy structures derived from base paring probabilities (Lorenz et al., 2011). The web version contains the most used tools and can be applied to obtain a putative secondary structure of a specific sequence or the consensus structure of a group of sequences (Hofacker, 2003). The Mfold algorithm uses free energy data to predict the minimum free energy for different foldings based on several user defined parameters. The output of Mfold includes structure plots, single strand frequency plots, and energy plots (Zuker, 2003). Another tool to predict secondary structure of RNAs is the Rfold algorithm which performs base paring probabilities (Kiryu et al., 2008).

**Table 7:** **Secondary structure prediction tools**.

Name	Site	Authors
Mfold	http://www.bioinfo.rpi.edu/applications/mfold	Zuker (2003)
ViennaRNA package	http://www.tbi.univie.ac.at/~ivo/RNA/	Lorenz et al. (2011)
Rfold	http://www.ncrna.org/software/Rfold/	Kiryu et al. (2008)

Other additional step in the interpretation of HTS ncRNA experiments includes finding the protein coding genes targeted by the detected ncRNAs. Even for the most studied ncRNA class, miRNAs, this is a complex task, due to their small size and few base pairing to their targets. The currently available tools rely on known properties like paring pattern, thermodynamic stability, and conservation to predict putative targets (Min and Yoon, 2010). There are several databases and software for miRNA target recognition (Table 8). Among them, may be cited Miranda (John et al., 2004), Pictar (Krek et al., 2005), and Diana-microT (Maragkakis et al., 2009; for a complete view of such databases, see, Yousef et al., 2009). The Miranda algorithm was used to predict miRNA targets presented in the microRNAs.org database (Betel et al., 2008). This algorithm uses the binding energy, complementary pattern, evolutionary conservation, and position of the binding site in the mRNA. Also, is the unique program which is available for download (John et al., 2004). The Pictar algorithm uses the type of paring between miRNA and mRNA, the free energy of the paring and target site conservation to generate a probability and a score of the putative target site (Krek et al., 2005). The DIANA-microT algorithm uses the type of paring and the conservation to calculate a score for each predicted binding site. This score is compared to the score obtained by using random miRNAs to calculate a signal-to-noise ratio (Maragkakis et al., 2009).

**Table 8:** **miRNA target prediction tools and databases**.

Name	Site	Description	Authors
TargetScan	http://www.targetscan.org/	miRNA target prediction algorithm	Lewis et al. (2003)
DIANA-microT	http://diana.cslab.ece.ntua.gr/microT/	miRNA target prediction algorithm	Maragkakis et al. (2009)
RNA Hybrid	http://bibiserv.techfak.uni-bielefeld.de/rnahybrid/	Tool for finding the minimum free energy hybridization of a long and a short RNA	Rehmsmeier et al. (2004)
miRDB	http://mirdb.org/miRDB/	Database for miRNA target prediction by MirTarget2 and functional annotation	Wang and El Naqa (2008)
microRNA.org	http://www.microrna.org/microrna/home.do	Database of miRNA target prediction by the miRanda algorithm	Betel et al. (2008)
TarBase	http://diana.cslab.ece.ntua.gr/tarbase/	Manually curated database of experimentally supported microRNA targets	Papadopoulos et al. (2009)
miR2Disease	http://www.mir2disease.org/	Manually curated database of miRNA deregulation in various human diseases	Jiang et al. (2009)
miRecords	http://mirecords.biolead.org/index.php	Database of experimentally validated miRNA targets and integration of predicted miRNA targets produced by 11 miRNA target prediction programs	Xiao et al. (2009)

The visualization of the reads aligned to the reference genome is another important set of tools for projects working with HTS. Data visualization permits to researchers to investigate HTS experiments in a user friendly way (Zhou et al., 2011). Several tools were developed for visualization of HTS experiments, some of them were listed by Lee et al. (2011), among them are Integrated Genomics Viewer (IGV; Thorvaldsdottir et al., 2012), Artemis (Carver et al., 2012), and Tablet (Milne et al., 2010). Also, the UCSC and Ensembl genome browsers have been updated to support HTS data. The downside of using a web viewer is uploading large amount of data (Fiume et al., 2010). Table 9 shows some bioinformatics tools for visualization of HTS experiments.

**Table 9:** **Tools for visualizations of HTS experiments**.

Name	Site	Authors
BamView	http://bamview.sourceforge.net/	Carver et al. (2012)
IGV	http://www.broadinstitute.org/igv/	Thorvaldsdottir et al. (2012)
Artemis	http://www.sanger.ac.uk/resources/software/artemis/	Carver et al. (2012)
Savant	http://genomesavant.com/savant/	Fiume et al. (2010)
Tablet	http://bioinf.scri.ac.uk/tablet/	Milne et al. (2010)

## Challenges in Bioinformatics of ncRNA and HTS

The management of the data produced by HTS methods is the first challenge in bioinformatics. Many gigabytes of raw data may be produced during a regular project aiming to detect the expression profile of ncRNAs in oncology and this amount may increase if it is considered data of mapped reads and all annotation databases used to analyze them. Furthermore, the hardware and network speed may be taken into account for appropriate analysis prior starting a HTS project. Other important challenge in Bioinformatics is to create protocols to assist in the analysis of ncRNA data. There are some efforts to assist protein coding genes in HTS data, but none was taken to ncRNA genes (Trapnell et al., 2012). Almost every article analyzing ncRNA expression profile using HTS methods present distinct normalization and statistical approaches. Finally, since Bioinformatics is still an emerging field of knowledge, there is few groups with graduate students developing innovative projects in bioinformatics and ncRNAs. In conclusion, there are three major limitations in bioinformatics of HTS projects: data management, analysis, and visualization; definition of protocols to data analysis; and professionals with expertise in ncRNA analysis.

## Conflict of Interest Statement

The authors declare that the research was conducted in the absence of any commercial or financial relationships that could be construed as a potential conflict of interest.
